# Suppressed competitive exclusion enabled the proliferation of Permian/Triassic boundary microbialites

**DOI:** 10.1002/dep2.97

**Published:** 2019-11-20

**Authors:** William J. Foster, Katrin Heindel, Sylvain Richoz, Jana Gliwa, Daniel J. Lehrmann, Aymon Baud, Tea Kolar‐Jurkovšek, Dunja Aljinović, Bogdan Jurkovšek, Dieter Korn, Rowan C. Martindale, Jörn Peckmann

**Affiliations:** ^1^ Museum für Naturkunde Leibniz Institute for Research on Evolution and Biodiversity Berlin Germany; ^2^ Institute for Earth and Environmental Sciences University of Potsdam Potsdam‐Golm Germany; ^3^ Jackson School of Geosciences University of Texas at Austin Austin TX USA; ^4^ Department of Geodynamics and Sedimentology University of Vienna Vienna Austria; ^5^ Department of Geology Lund University Lund Sweden; ^6^ Institute of Earth Sciences Graz University Graz Austria; ^7^ Geosciences Department Trinity University San Antonio TX USA; ^8^ Institute of Earth Sciences Lausanne University Lausanne Switzerland; ^9^ Geological Survey of Slovenia Ljubljana Slovenia; ^10^ Faculty of Mining, Geology and Petroleum Engineering University of Zagreb Zagreb Croatia; ^11^ Department of Geological Sciences University of Texas at Austin Austin TX USA; ^12^ Institut für Geologie Centrum für Erdsystemforschung und Nachhaltigkeit Universität Hamburg Hamburg Germany

**Keywords:** Competitive exclusion, Permian/Triassic, mass extinction, microbialites, palaeoecology

## Abstract

During the earliest Triassic microbial mats flourished in the photic zones of marginal seas, generating widespread microbialites. It has been suggested that anoxic conditions in shallow marine environments, linked to the end‐Permian mass extinction, limited mat‐inhibiting metazoans allowing for this microbialite expansion. The presence of a diverse suite of proxies indicating oxygenated shallow sea‐water conditions (metazoan fossils, biomarkers and redox proxies) from microbialite successions have, however, challenged the inference of anoxic conditions. Here, the distribution and faunal composition of Griesbachian microbialites from China, Iran, Turkey, Armenia, Slovenia and Hungary are investigated to determine the factors that allowed microbialite‐forming microbial mats to flourish following the end‐Permian crisis. The results presented here show that Neotethyan microbial buildups record a unique faunal association due to the presence of keratose sponges, while the Palaeotethyan buildups have a higher proportion of molluscs and the foraminifera *Earlandia*. The distribution of the faunal components within the microbial fabrics suggests that, except for the keratose sponges and some microconchids, most of the metazoans were transported into the microbial framework via wave currents. The presence of both microbialites and metazoan associations were limited to oxygenated settings, suggesting that a factor other than anoxia resulted in a relaxation of ecological constraints following the mass extinction event. It is inferred that the end‐Permian mass extinction event decreased the diversity and abundance of metazoans to the point of significantly reducing competition, allowing photosynthesis‐based microbial mats to flourish in shallow water settings and resulting in the formation of widespread microbialites.

## Graphical abstract

After the end‐Permian mass extinction, microbialites filled the ecological niche previously occupied by metazoan reefs. The factors that allowed microbialite‐forming microbial mats to flourish are, however, hotly debated. By investigating the faunal composition and depositional setting of Permian/Triassic boundary microbialites, we propose that the impact of the extinction event on the abundance of metazoans suppressed the biological controls that were previously excluding microbialite development from subtidal environments.

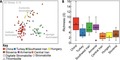

## 1 INTRODUCTION

Some biotic crises and mass extinction events are associated with the globally widespread replacement of skeletal carbonates with microbialites (Schubert and Bottjer, 1992; Baud *et al.*, 1997, 2007; Pruss and Bottjer, 2004; Sheehan and Harris, 2004; Sremac *et al.*, 2016; Yao *et al.*, 2016). Microbialite proliferation following the end‐Permian mass extinction—the most catastrophic extinction event of the Phanerozoic—resulted in the development of thick microbialite successions and reefal microbial buildups, which covered large areas of the Early Triassic ocean margins (Kershaw *et al.*, 2012; Martindale *et al.*, 2019). The circumstances that allowed microbialite‐forming microbial mats to flourish and dominate post‐extinction successions are, however, poorly understood. For Precambrian microbialites, the upwelling of anoxic, alkaline waters onto the shelf was hypothesized to have led to conditions that were greatly supersaturated with respect to calcium carbonate, favouring widespread microbialite development (Grotzinger and Knoll, 1995). In the case of the Precambrian, deep waters of stratified seas were posited to have been anoxic and carbonate alkalinity was supposedly increased by bacterial sulphate reduction. This model of microbialite development has also been applied to the development of microbialites in the Early Triassic and has been used to explain the end‐Permian mass extinction event (Kershaw *et al.*, 1999, 2018; Pruss *et al.*, 2006; Mata and Bottjer, 2011, 2012).

The presence of metazoans intolerant of low‐oxygen conditions within the Early Triassic microbialites has challenged this view and suggests that microbialites formed under oxic conditions (Wignall and Twitchett, 2002; Marenco *et al.*, 2012; Tang *et al.*, 2017) or that metazoans lived in a biological refuge facilitated by microbial mats (Forel *et al.*, 2013b). The upwelling of anoxic water hypothesis has also been challenged for the Early Triassic because one expectation is that microbialite successions on the edge of isolated platforms with greater exposure to upwelling should hypothetically be thicker than on the platform interior, which is not consistent with observations from China (Lehrmann *et al.*, 2015). Clearly, evaluating the mechanisms responsible for the high abundance of microbialites during the Early Triassic remains a source of considerable debate (Bagherpour *et al.*, 2017; Friesenbichler *et al.*, 2018).

**Figure 1:**
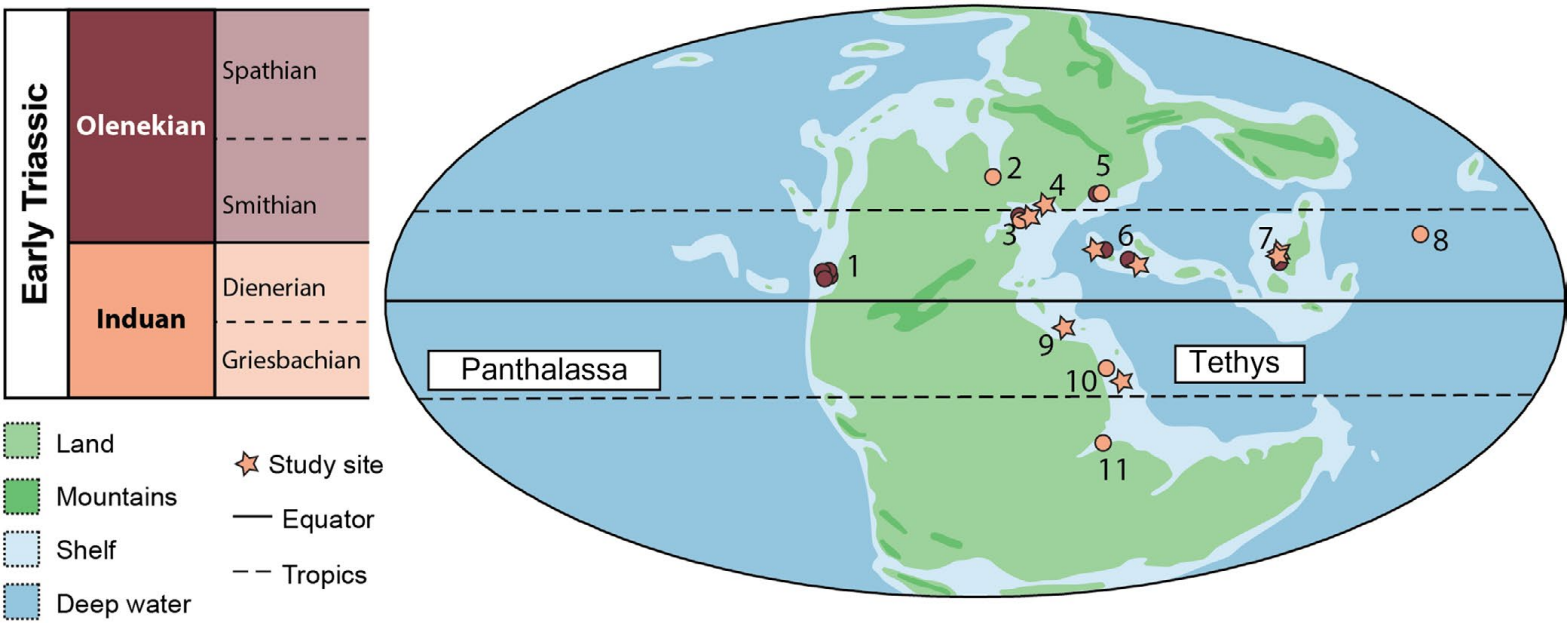
Palaeogeographical map showing the location of known microbialite successions following the end‐Permian mass extinction. 1: Nevada and Utah (USA), 2: Greenland, 3: northern Italy and Slovenia, 4: Hungary, 5: Caucasus, 6: central Iran and Armenia, 7: South China and Vietnam, 8: southern Japan, 9: southern Turkey, 10: southwest Iran, UAE and Oman, 11: Madagascar. Palaeomap after Golonka (2002) and the location of significant microbialite successions (i.e., biostromes and reefs) modified after Martindale et al. (2019). Circles correspond to localities known to record microbialites but not sampled in this study. Colours correspond to the different ages of the microbialites

**Figure 2:**
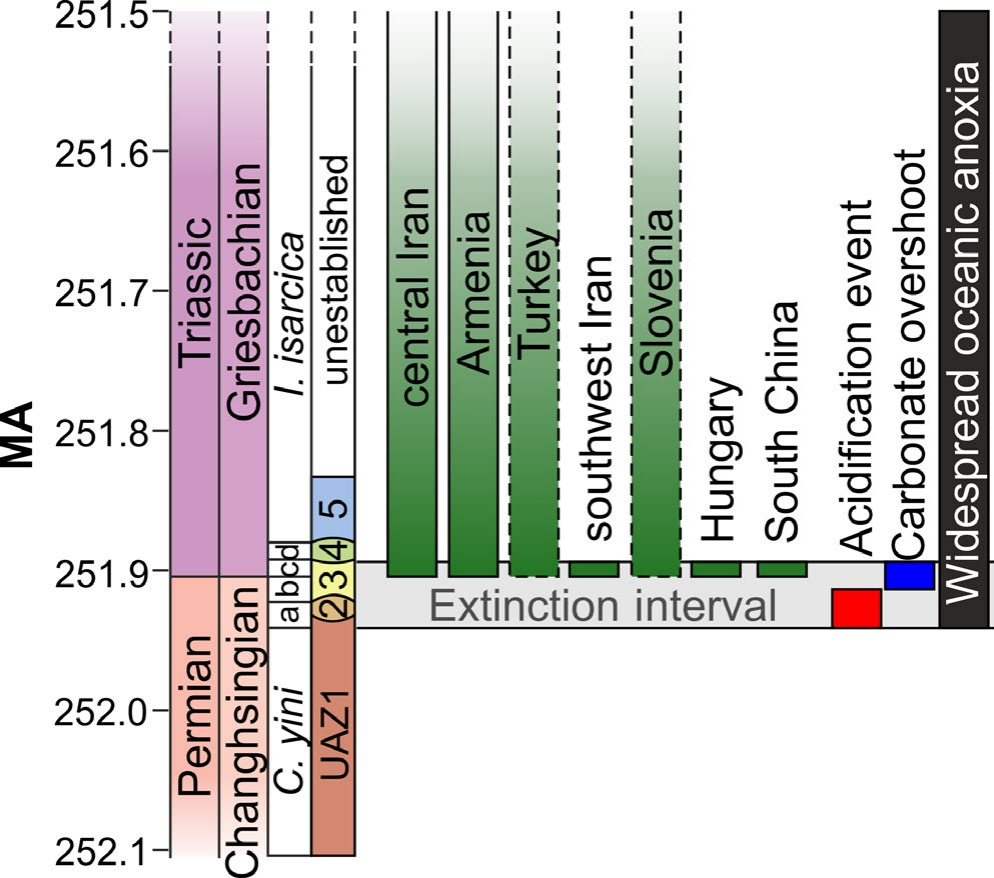
Stratigraphic correlation of the microbialite successions investigated in this study and the development of the hypothesized drivers of extinction. The microbialites investigated in this study are symbolized by the vertical green bars. The hypothesized duration of ocean acidification and timing of a subsequent carbonate overshoot after cGENIE Earth‐system model (Cui *et al*., 2013). The duration of widespread oceanic anoxia is based on the Permian–Triassic uranium^238^ isotope perturbation after Brennecka *et al*. (2011) and Lau *et al*. (2016). Data for the correlation of microbialites is discussed in the text and shown in the stratigraphic logs for each section in Figure S1. Radiometric ages are based on U/Pb dates after Burgess *et al*. (2014). Conodont biostratigraphy from the P/Tr boundary GSSP after Yuan *et al*. (2014): *C. yini* = *Clarkina yini*, a = *C. meishanensis*, b = *H. changxingensis* – *C. zhejiangensis*, c = *Hindeodus parvus*; d = *Isarcicella staeschei,* and conodont unitary associations (UAZ1 to 5) after Brosse *et al*. (2016). Extinction interval is shown as a horizontal grey bar following Wang *et al*. (2014)

Reported here are the results of an investigation into the spatial and faunal composition of microbialite successions from China, Iran, Turkey, Armenia, Slovenia, and Hungary (Figure 1). Focus was placed on Tethyan microbialites that formed at the base of the Triassic (Griesbachian substage) from the *Hindeodus parvus* to the *Isarcicella isarcica* conodont zones (except in the Chanakhchi Hill (Armenia) and Baghuk sections (central Iran), which developed from the *H. parvus* to *Sweetospathodus kummeli* Zones) (Figure 2). This work also builds on previous detailed stratigraphic, geographic and depositional frameworks that have been developed for these locations (Baud *et al.*, 1997, 2005, 2007; Richoz, 2006; Hips and Haas, 2006; Insalaco *et al.*, 2006; Richoz *et al.*, 2010; Kershaw *et al.*, 2011; Leda *et al.*, 2014; Lehrmann *et al.*, 2015; Kolar‐Jurkovšek *et al.*, 2018; Friesenbichler *et al.*, 2018) to investigate the environmental conditions that permitted the development of globally widespread microbialites during the Early Triassic.

## 2 STUDY SECTIONS AND METHODS

Twenty‐one stratigraphic sections with microbialites that developed immediately following the end‐Permian mass extinction event were logged and sampled; these include sections in China (Great Bank of Guizhou: Youjianzhai, Xingbaihe, Dongjiawen, Houchang, Dawen, Dajiang, Longbai, and Rongbao sections), south‐west Iran (Zagros Mountains: Kuh‐e‐Surmeh and Kuh‐e‐Dena sections), central Iran (Shahreza and Baghuk Mountain sections), Turkey (Çürük Dag and Oznur Tepee sections), Armenia (Chanakhchi Hill and Ogbin sections), Slovenia (Masore, Idrijca, and Vojsko sections) and Hungary (Balvány‐North and Balvány‐East sections); see Figure S1 for logs of these sections. A total of 125 large thin sections were made from hand samples. The 48 samples from the Great Bank of Guizhou (China) were made into polished slabs.

Bioclasts were counted by placing an acetate sheet that had been divided into 5 mm square divisions over the entire thin section of each sample. All the bioclasts within each quadrant were identified to measure taxonomic richness and tallied to obtain abundance data. In this study, taxonomic identifications are mostly confined to class‐level, comparable to Early and Middle Triassic investigations (Payne *et al.*, 2006; Jacobsen *et al.*, 2011; Yang *et al.*, 2011). The samples varied in size, with the number of 5 mm square divisions ranging from 26 to 422 squares. Nevertheless, a cross plot between sample size and species richness (Figure S2) shows that sample size did not affect the observed richness.

Palaeoecological analyses were limited to benthic marine invertebrates and foraminifera. Volume (i.e., the number of squares each taxon occurred in) was used to calculate relative abundances, as this allows different groups of animals that develop differently (i.e., solitary vs. colonial organisms) and organisms of different sizes to be compared fairly. The Kruskal–Wallis test was used to statistically test the differences in the median diversity between different groups of samples. A non‐metric multidimensional scaling (nMDS) was then applied to visualize trends and groupings of the samples. For the nMDS, samples with no bioclasts were removed because such samples would have zero similarity to fossiliferous samples and would plot randomly in an ordination. Relative, rather than absolute, abundances were used, as preservation varies between samples. Most of the samples are dominated by a few taxa, and so the relative abundance data were square‐root transformed to de‐emphasize the influence of the most dominant taxon. A Bray–Curtis similarity matrix was then applied to recognize those taxa that tend to co‐occur in samples of similar taxonomic compositions. Statistical analysis and visualization was done using the ‘vegan’, and ‘ggplot2’ packages in R (R Core Team, 2017).

## 3 GEOLOGICAL AND PALAEOENVIRONMENTAL SETTING

**Figure 3:**
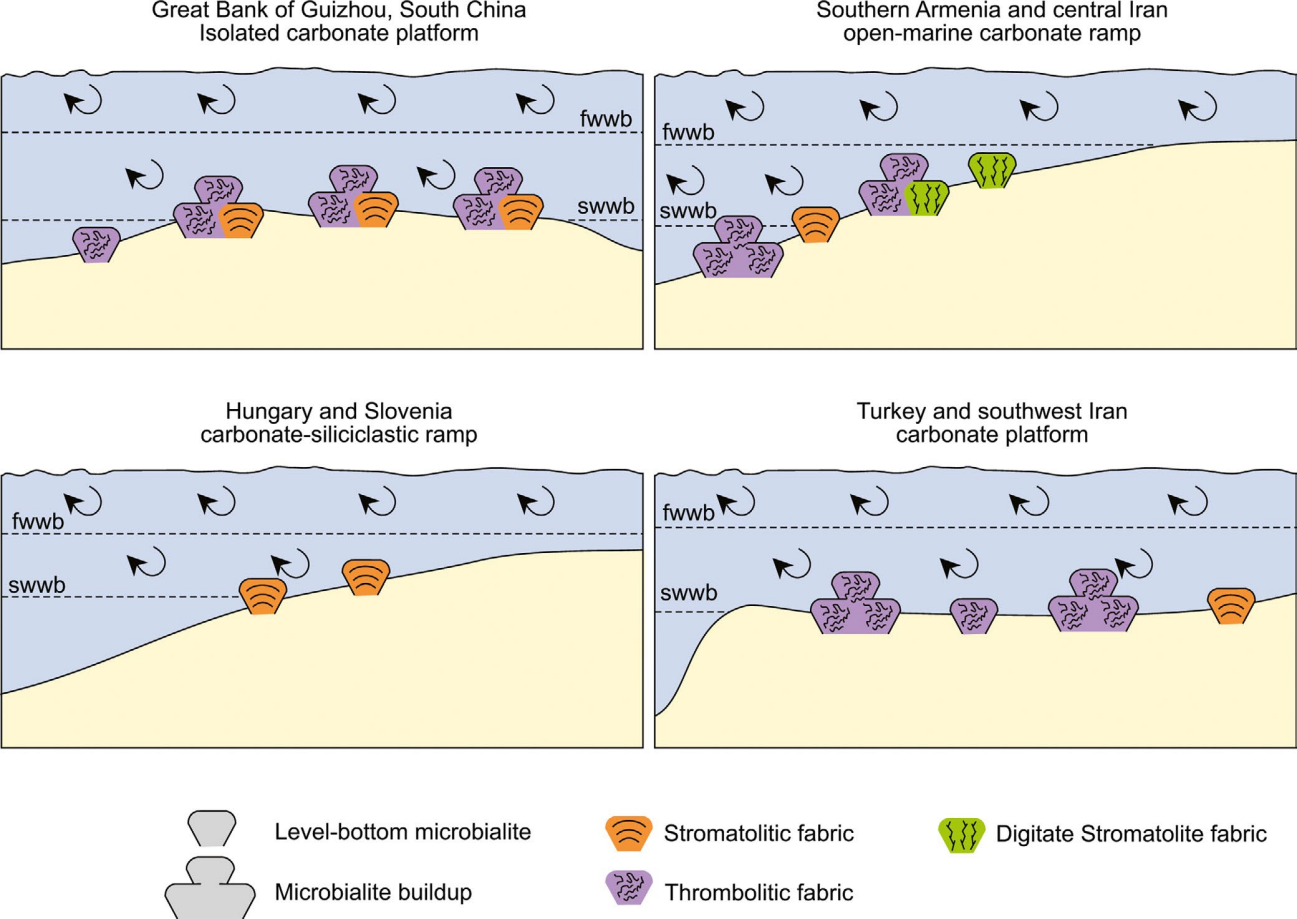
Schematic showing the palaeoenvironmental distribution of the Permian/Triassic boundary microbialites. fwwb = fair weather wave base, swwb = storm weather wave base. Arrows indicate wave‐driven ocean circulation

The Great Bank of Guizhou, China, is an exceptionally well‐exposed ~600 km^2^ isolated platform that formed in the Nanpanjiang Basin in the eastern Palaeotethys. Due to the platform being exhumed with its depositional profile preserved, the mechanisms involved in its development can be reconstructed from numerous sections (Lehrmann *et al.*, 1998). The Great Bank of Guizhou is one of many isolated platforms known from the Nanpanjiang Basin, but records one of the thickest Griesbachian microbialite successions (up to 19 m thick; Figure S1). The microbialites that make up the succession can be characterized broadly as one of three types: both stromatolite or thrombolite biostromes and isolated thrombolitic domes with individual thicknesses that range from a few centimetres up to 1.5 m (Figure 3). There are also localized domes within the biostromes that formed small topographic highs on the seafloor. The microbial buildups on the Great Bank of Guizhou occur within a narrow range of environmental conditions. The thickest microbialite successions occur on the inner platform and are intercalated with oolitic and skeletal packstones and grainstones that contain a diverse, para‐autochthonous assemblage (Foster *et al.*, 2019a). Cross‐bedding can be recognized in the grainstones that drape over the microbialites. In addition, disarticulated shells are randomly orientated within the microbialites, and are interpreted to have been transported in a storm wave‐agitated environment. In contrast, in sections that were closer to the platform margin, such as at Rongbao, the microbialite unit is thinner and is interpreted to have formed in deeper (and thus quieter) settings, closer to storm weather wave base. The distribution of microbialites from the *H. parvus* Zone on the Great Bank of Guizhou also differ to the younger Dienerian microbialites, which are limited to shallower settings on the lower part of an intertidal flat (Lehrmann *et al.*, 2001).

In sections distal to the Great Bank of Guizhou, which record deposition well below wave base, microbialites have not been recognized (Lehrmann *et al.*, 1998; Yan *et al.*, 2013). In these sections, bottom‐water anoxia is indicated by laminated shales, geochemical proxies (i.e., thorium/uranium ratios, positive cerium anomalies; Song *et al.*, 2012), and the presence of the dysoxia‐tolerant bivalve *Claraia* (Lehrmann *et al.*, 1998; Huang *et al.*, 2018). In addition, the development of microbialites on the Great Bank of Guizhou temporarily ceased (i.e., at the top of the basal microbialite unit) following a sea‐level rise and deepening of the settings below wave base. This has also been recognized across the whole of the Nanpanjiang Basin (Kershaw *et al.*, 2012; Bagherpour *et al.*, 2017), with the overlying deposits being made up of laminated carbonate mudstones and laminated brown siltstones interpreted to reflect deposition in a low‐energy, anoxic setting. In some sections, deposition above storm weather wave base was restored within a few metres, but the formation of microbialites in shallow subtidal settings was replaced by thick oolitic shoals (e.g. Houchang), or skeletal packstones (e.g. Heping). This suggests that the environmental conditions favourable for the formation of widespread microbial frameworks in shallow subtidal environments were no longer present on the Great Bank of Guizhou after the *H. parvus* Zone.

**Figure 4:**
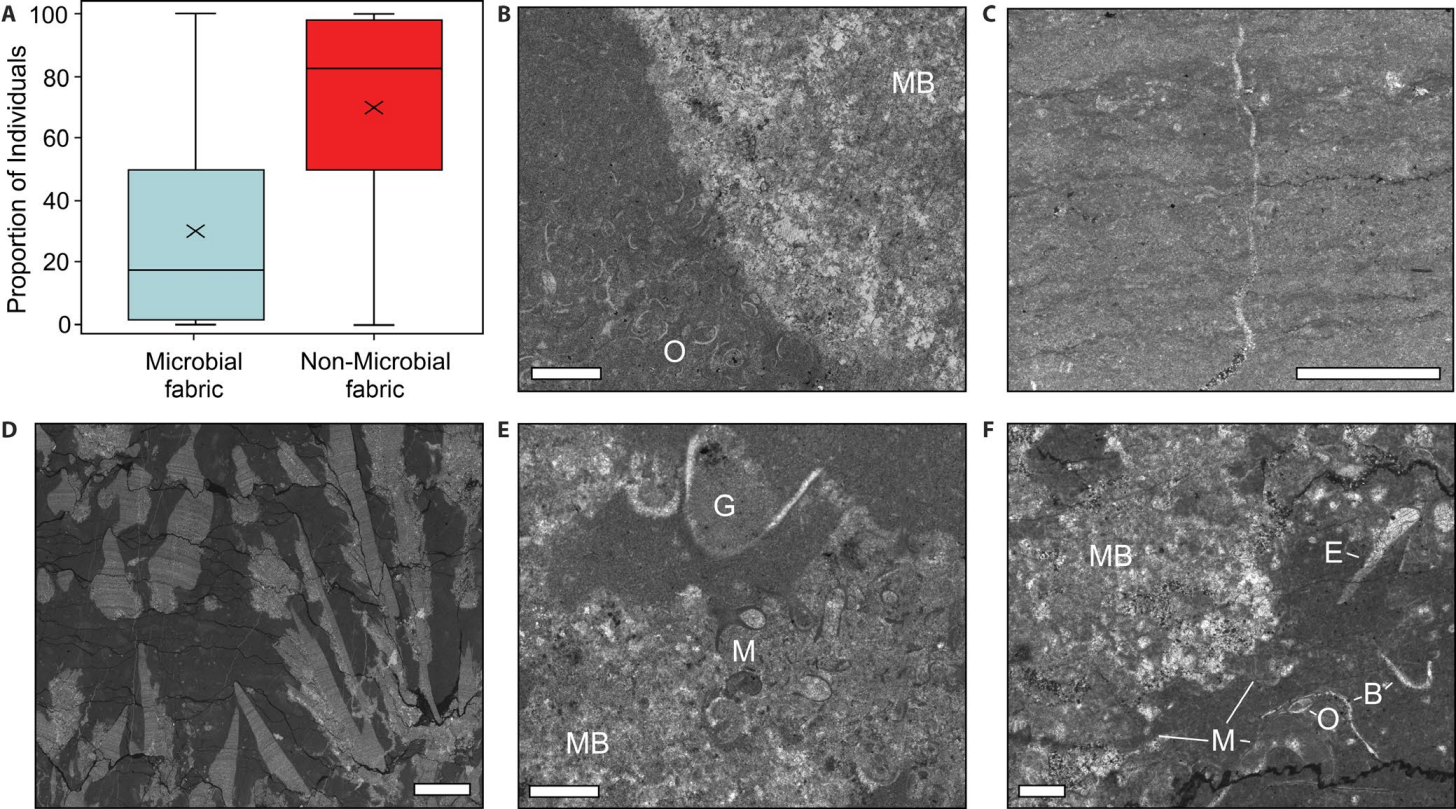
Substrate selectivity of metazoans in the Permian/Triassic boundary microbialites. (A) Comparison of the number of bioclasts recorded from the microbial and non‐microbial fabrics of the studied samples. The solid black lines inside the boxes represent the medians, the x inside the boxes show the mean, the top and bottom edges of the boxes correspond to the first and third quartiles, and whiskers represent the lowest and highest datum within 1.5 times the interquartile range. Only samples with both microbial and non‐microbial fabrics are included. (B–F) Examples of microbialite fabrics and distribution of fossils in microbialite samples. (B) Accumulation of ostracods (O) in micrite surrounding the thrombolitic fabric (MB) from the Zagros Mountains, south‐west Iran. (C) Microbial laminae from the Bükk Mountains, Hungary, with rare fossils. (D) Digitate stromatolite from Shahreza, central Iran. (E) Accumulation of reworked microconchids (M) in the thrombolite fabric (MB) and a gastropod (G) in the surrounding micrite, Zagros Mountains, south‐west Iran. (F) Randomly orientated bivalves (B), echinoids (E), ostracods (O) and microconchids (M) in the micrite surrounding the thrombolitic fabric (MB), Great Bank of Guizhou, South China. Scale bars = 1 mm, except in C, which is 0.5 mm

The microbial buildups in Çürük Dag (Turkey), Kuh‐e‐Surmeh, and Kuh‐e‐Dena (Zagros, south‐west Iran) also represent deposition above storm wave base, but on a platform setting on the south‐west margin of the Neotethys Ocean (Figures 1 and 3B). The microbialite unit at Çürük Dag is 14 m thick and is represented by stromatolites and thrombolites, which are intercalated with oolitic packstones and grainstones (Figure S1). The relief of individual microbial‐metazoan bioherms reaches up to ~2.5 m. Their deposition is interpreted to have occurred around fair weather wave base (Baud *et al.*, 2005; Kershaw *et al.*, 2011). At Kuh‐e‐Surmeh the microbialites are exclusively thrombolitic, whereas at Kuh‐e‐Dena they are mainly digitate stromatolites or thrombolitic. At Kuh‐e‐Surmeh and Kuh‐e‐Dena the microbialites are ~3 and ~6 m thick, respectively. At Kuh‐e‐Dena there are interlayers in between microbial bioherms (up to 1.5 m), with hummocky cross‐stratified grainstones, which indicates a depositional setting above storm wave base. The fauna within the microbial buildups also appears to be transported and concentrated in the surrounding matrix and in cavities of the microbial buildups (Figure 4). Based on conodont stratigraphy, the microbialites in the Zagros Mountains are older than at Çürük Dag, with the former developing during the *H. parvus* Zone, whereas in Çürük Dag, the thickness of the *H. parvus* Zone is reduced to only a few centimetres and the microbialites are from the mid‐Griesbachian *I. isarcica* Zone (Richoz, 2006; Heindel *et al.*, 2018). Carbon isotope profiles of the Zagros Mountains and Çürük Dag sections, however, suggest that deposition of microbialites is contemporaneous in both regions (Richoz, 2006; Richoz *et al.*, 2010) and, consequently, *I. isarcica* may have appeared earlier in Çürük Dag than in other microbialite bearing sections.

The carbonate lithologies studied in Hungary and Slovenia were deposited on a vast mixed carbonate‐siliciclastic ramp in the western Palaeotethys, and are interpreted to have been deposited around or below storm wave base (Hips and Haas, 2006; Aljinović *et al.*, 2018; Kolar‐Jurkovšek *et al.*, 2018). The stromatolites found in Hungary and Slovenia are submillimetre alternations of putative microbial precipitates (dense or clotted micrite laminae) and homogenous, finely crystalline (microsparitic) laminae, possibly detrital in origin. Thus, the succession of inferred microbial precipitates is punctuated by detrital carbonate laminae or interlayers. Detrital components commonly fill the space between the dense micritic elements, or loose, reworked microbial fragments are contained in the detrital laminae. The detrital laminae also contain randomly oriented ostracod, foraminifera, bivalve and gastropod fossils. Thus, winnowing of microbial mats by constant weak currents has been inferred (Hips and Haas, 2006; Kolar‐Jurkovšek *et al.*, 2018). In addition, the distribution of the fossils, which is confined to the thin detrital laminae, suggest an allochthonous origin and reflects offshore transportation of bioclasts by weak storm currents around storm wave base. These lithologies developed during the *Hindeodus praeparvus* and *H. parvus* zones (both in Hungary and Slovenia) and in Slovenia extend up through the *I. isarcica* Zone, associated with the mass extinction event (Sudar *et al.*, 2008; Kolar‐Jurkovšek *et al.*, 2018). In the Bükk Mountains (Hungary), the laminated carbonate rocks are overlain by a thick mudstone unit that is interpreted to represent a transgression, resulting in deeper, low‐energy, dysoxic or anoxic settings (Hips and Haas, 2006). The microbialite beds disappear in this interval, and, therefore, it is believed that the parent microbial mats grew near the limit of their environmental tolerance.

The central Iran and Armenia sections represent deposition on the western margin of the Cimmerian microcontinent between the Neotethys and Palaeotethys oceans (Figure 1). These sections are characterized by a non‐calcareous ‘boundary clay’ that grades into a ‘boundary marl’ that formed immediately after the mass extinction event (Richoz *et al.*, 2010; Ghaderi *et al.*, 2014; Schobben *et al.*, 2016). The boundary clay is overlain by platy lime mudstones that intercalated with microbialites and abiotic crystal fans (Figure S1), representing deposition on a low‐relief distal carbonate platform below fair weather wave base (Figure 3).

In the Armenian section, microbialites are particularly abundant and range in size from small domes a few centimetres in thickness to large domes up to 4 m in height in the Ogbin section and 12 m in the Chanakhchi Hill section (see also Friesenbichler *et al.*, 2018). Conodont stratigraphy revealed that the microbialites formed from the earliest Griesbachian *H. parvus* to the Dienerian *S. kummeli* conodont Zone (Zakharov *et al.*, 2005), but the larger structures formed during the Griesbachian (Zakharov *et al.*, 2005). Towards the upper part of the microbialite succession, packstones become more abundant, containing relatively large bellerophontids larger than 10 mm.

In the Shahreza and Baghuk sections (central Iran), the microbialites are thinner than in Armenia, and do not exceed 2 m in height (Baud *et al.*, 2007) (Figure S1). Conodonts are only known from the basal 4 m of the Shahreza section, which indicates that the lower microbialite developed from the post‐extinction *H. praeparvus* to *I. isarcica* conodont zones (Kozur, 2005; Richoz *et al.*, 2010), whilst the microbialites that developed at 8 and 19 m in the Shahreza section likely developed in the middle to late Griesbachian. The microbialites from the Baghuk section have previously been described as poorly structured thrombolites and cryptic microbialites (Leda *et al.*, 2014), but are dominantly structures made up of columns of radial crystal fans. In addition, radial crystal fan structures that form ‘mushroom’ shapes occur in association with small (~1 cm) columnar stromatolites, but whether the mushroom structures have a microbial origin is equivocal. The microbialites from Ogbin, Chanakhchi, Shahreza and Baghuk all differ in composition from microbialites from the Great Bank of Guizhou, Zagros Mountains, Çürük Dag, Hungary and Slovenia in having a higher proportion of digitate stromatolites and crystal fans. Furthermore, small mounds just above the extinction horizon at the Baghuk Mountain, Shahreza, Ogbin and Chanakhchi sections are exclusively made up of digitate stromatolites and radial crystal fans.

## 4 FAUNAL COMPOSITION OF POST‐EXTINCTION MICROBIALITES

**Figure 5:**
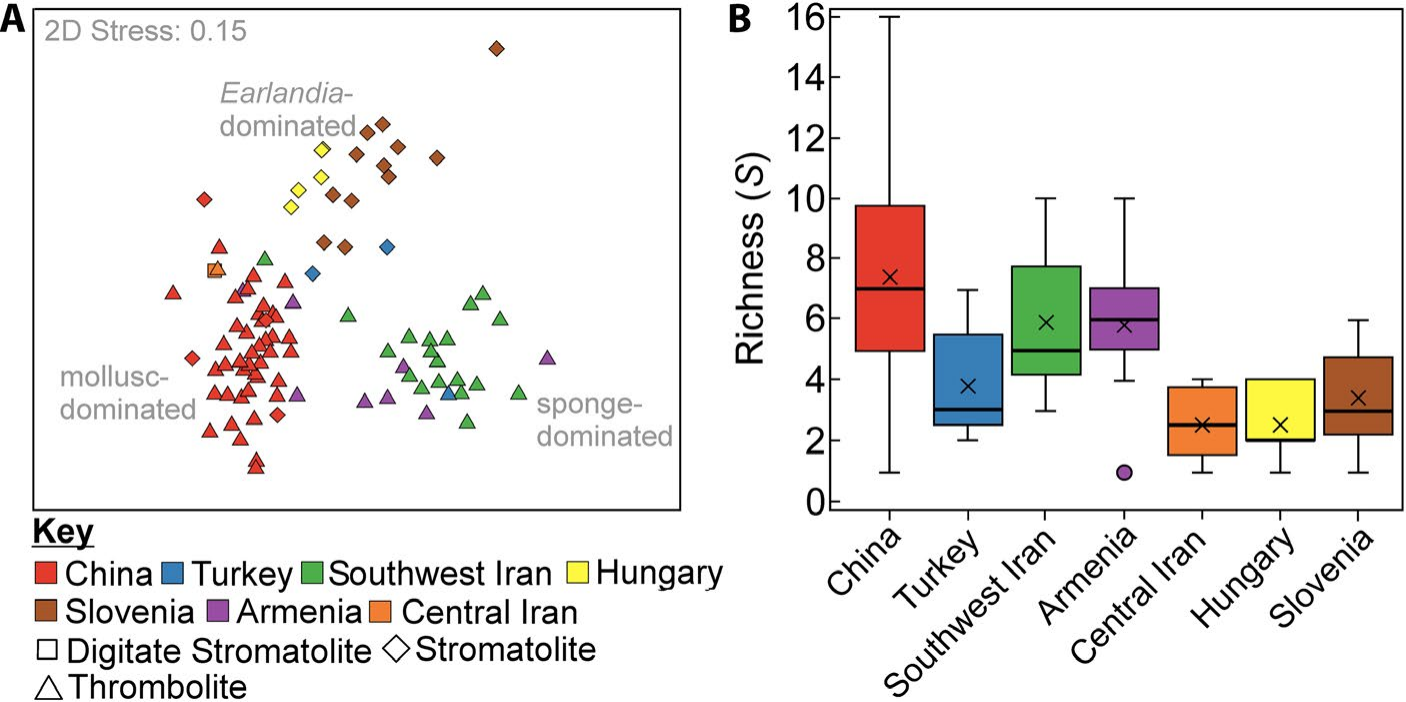
Faunal composition of microbialites following the end‐Permian mass extinction. (A) Non‐metric multidimensional scaling plot (nMDS) of each microbialite sample based on a Bray–Curtis similarity matrix. (B) Box plots showing the taxonomic richness of samples in each of the investigated regions. See Figure 4 for an explanation of box plots; the dots outside the boxes are outliers

In all of the investigated localities, independent of region and stratigraphic level, the microbialites contain a high‐abundance assemblage of ostracods, microconchids, gastropods, bivalves and foraminifera, which have already been previously recognized (Baud *et al.*, 1997; Forel *et al.*, 2009, 2013a; Yang *et al.*, 2011; Foster *et al.*, 2018). These results show significant separation between the different investigated regions with the samples plotting into three main groups (Figure 5A). The faunal composition of the microbialites in southwest Iran, Armenia, and Turkey, representing Neotethyan sections, are dominated by keratose sponges and, to a lesser extent, by microconchids, ostracods and the agglutinated foraminifera *Earlandia* (Dataset S1). In the field, keratose sponges are also observed as being abundant in central Iran (Shahreza and the Baghuk Mountains). Where the keratose sponges are absent, the Neotethyan samples display a closer relationship to the Great Bank of Guizhou assemblages dominated by ostracods, gastropods, microconchids and bivalves. The Great Bank of Guizhou microbialites also contain abundant echinoid spines, which appear to be unique to China. They are also significantly more diverse than the microbialites elsewhere (*p* < 0.05; Figure 5B).

The third distinct group includes the microbialite‐metazoan associations from Hungary and Slovenia (Figure 5A). The diversity of these associations is also significantly less than the microbialite successions found elsewhere, except for central Iran (Figure 5B). In addition, >80% of the fossil assemblage is dominated by ostracods and *Earlandia*; thus, the biogenic composition of these samples is relatively homogeneous. Despite the microbialites being relatively diverse when compared to some contemporaneous level‐bottom successions, for example, Mazzin Member, Italy (Foster *et al.*, 2017), these faunas are still impoverished and record small sizes (typically <2 mm). It is the small size of each individual contributor that allows a high concentration of fossils to be recorded in the microbialite samples. In addition, except for the keratose sponges, the fossils are randomly orientated, disarticulated and commonly concentrated in the micrite surrounding the microbial fabric (Figure 4). This pattern is interpreted to reflect winnowing of fossils into the microbialites, rather than *in situ* preservation.

## 5 DISCUSSION

It has been suggested that the upwelling of anoxic water into shallow subtidal settings drove the end‐Permian mass extinction event, excluding some metazoans, and created alkaline conditions allowing microbial mats to flourish (Kershaw *et al.*, 1999, 2007; Pruss *et al.*, 2006; Mata and Bottjer, 2011). Furthermore, it has been suggested that because comparatively high‐diversity Griesbachian assemblages occur in the absence of microbialites that microbialites are restricted to anoxic environments (Kershaw *et al.*, 2007). The upwelling of anoxic‐waters scenario was also favoured because anoxic waters in stratified seas and lakes record elevated bicarbonate‐rich conditions due to bacterial sulphate reduction—a mechanism that has also been inferred for Early Triassic oceans (see Schobben *et al.*, 2015)—and ammonia formation (Grotzinger and Knoll, 1995; Sun *et al.*, 2019), which would have favoured microbialite development through an increase in seawater alkalinity.

The upwelling of alkaline waters onto the shelf is not thought necessary for the development of microbialites after the mass extinction event. The microbial communities that formed the microbialites created local carbonate supersaturation (Friesenbichler *et al.*, 2018) or favoured mineralization by interactions of solutes with bacterial extracellular polymeric substances (Dupraz *et al.*, 2009), allowing the lithification of microbial mats and microbialite development in the absence of critically supersaturated seawater (Riding, 2006). Furthermore, shortly after the end‐Permian mass extinction, an ‘alkalinity overshoot’ would have driven carbonate saturation states up. The alkalinity overshoot would have resulted from the large volumes of carbon dioxide emitted into the atmosphere (Kump *et al.*, 2009) by the Siberian Traps volcanism; elevated carbon in the atmosphere led to dissolution of carbonate sediment and the retention of dissolved carbonate in seawater, which subsequently resulted in the development of carbonate supersaturation following the Permian/Triassic (P/Tr) boundary (Cui *et al.*, 2015) (Figure 2). Alternatively, the elimination of the abundant skeletal carbonates during the mass extinction may have also caused or contributed to a rise in seawater alkalinity (Zhuravlev and Wood, 2009) that would have favoured the lithification of microbial mats.

Despite the inference of anoxic conditions, Early Triassic microbialites are associated with relatively high‐diversities of benthic invertebrates intolerant of low‐oxygen conditions, for example, ostracods and echinoderms (Yang *et al.*, 2011; Marenco *et al.*, 2012; Forel *et al.*, 2013b; Hautmann *et al.*, 2015; Pietsch *et al.*, 2016; Foster *et al.*, 2018) (Figure 5). The presence of such obligate aerobes was not used to falsify anoxic conditions, but instead, the microbial mat communities that precipitated the buildups were interpreted as unique oxygenated refugia in otherwise anoxic settings; this is known as the microbialite refuge hypothesis (Forel *et al.*, 2013b). Even though microbial mats can provide oxygenated refugia in hypoxic and anoxic environments (Gingras *et al.*, 2011), the fauna that occurs in the Early Triassic microbialites are mostly not animals that can live within a microbial mat (Foster *et al.*, 2019a). In addition, the fossils within the microbialites are allochthonous and have been transported around and into the microbial frameworks, thus the animals did not apparently live within the microbial mats (Foster *et al.*, 2019b) (Figure 4). Together, these observations allow for a rejection of the hypothesis that the microbialites were oxygenated refugia in an otherwise anoxic setting following the end‐Permian mass extinction event.

Contrary to this evidence, pyrite framboid size‐frequency distributions have been used to infer dysoxic water column conditions during the development of P/Tr boundary microbialites in South China (Liao *et al.*, 2010, 2017; Wang *et al.*, 2016). The size‐range of pyrite framboids recorded from the Early Triassic microbialites does not, however, indicate dysoxic water column conditions as similar‐sized pyrite framboids can develop due to microbial processes within the sediment (Schieber, 2002) and can occur within microbialite‐forming microbial mats (Cavalazzi *et al.*, 2007; Heindel *et al.*, 2012). The co‐occurrence of mostly allochthonous fossils and the microbialites also does not necessarily reject the hypothesis that microbialites formed in dysoxic–anoxic conditions, as the metazoans would not have lived in the same habitat. Redox proxies from the Griesbachian microbialite successions, that is, redox‐sensitive trace metals (Loope *et al.*, 2013; Collin *et al.*, 2014), lipid biomarkers (Chen *et al.*, 2011; Heindel *et al.*, 2018), and both total organic carbon and total sulphur contents (Tang *et al.*, 2017), however, have also been used to infer oxygenated sea‐water conditions. Consequently, these redox proxies also suggest that pyrite framboid size distributions in Early Triassic microbialites cannot unequivocally be used to infer water column conditions. Early Triassic microbialites, therefore, likely developed in marine settings with an oxygenated water column, which explains why microbialites and metazoans co‐occur in shallow subtidal settings.

The microbialites from South China, southwest Iran, Turkey (all this study) and Japan (Sano *et al.*, 1997) are restricted to wave agitated settings, signifying that oxygenation of the seafloor by waves promoted the diverse metazoan community associated with microbialite‐forming microbial mats. The distribution of microbialites on the wave‐aerated seafloor would have been locally and regionally variable depending on factors such as dissolved inorganic carbon, sedimentation rates, light penetration and grazing. In contrast, the microbialites investigated in Hungary, Slovenia, Armenia and central Iran occur in deeper settings close to storm weather wave base. In Hungary and Slovenia, the fossils are concentrated along thin laminae, and as sea‐level rose the microbial laminae and fauna disappeared (Hips and Haas, 2006). This, therefore, implies that even though the microbialites contain a diverse fauna, storm‐induced currents transported and introduced the allochthonous fauna to the microbialites. In Armenia and central Iran, there is currently no evidence for the development of anoxic conditions, and oxygenated bottom currents on the homoclinal platform have been proposed for the Griesbachian (Leda *et al.*, 2014; Friesenbichler *et al.*, 2018). The restriction of microbialites to shallow water environments is inferred to represent the restriction of the microbial communities to the photic zone. Primary production in the composite microbial mats was driven by oxygenic photosynthesis, which is consistent with lipid biomarkers and putative fossils of cyanobacteria (Yang *et al.*, 2011; Wu *et al.*, 2014; Heindel *et al.*, 2018).

Other environmental stressors that would have suppressed mat‐inhibiting metazoans in shallow marine environments include periods of high salinity (Heindel *et al.*, 2018), eutrophication (Meyer *et al.*, 2011; Schobben *et al.*, 2015) and high temperatures (Sun *et al.*, 2012; Schobben *et al.*, 2014). Despite these stressors, shallow marine environments record relatively high‐diversity communities following the mass extinction event (Figure 5) and thus it is inferred that environmental stressors did not reach lethal levels for all metazoans. Conversely, some of the relatively abundant metazoans within the microbialites include grazers (e.g., bellerophontids), which suggests that mat‐inhibitors were present, but not at microbialite suppressing abundances. The delayed recovery of metazoans following the end‐Permian mass extinction might also be a consequence of the magnitude of species loss itself, whereby the initial recovery of competition‐stimulated diversification rates and the sedimentary mixed‐layer was delayed due to reduced competition (Erwin, 2001; Pruss *et al.*, 2006; Hautmann *et al.*, 2015; Hofmann, 2016).

It is hypothesized here that the impact of the extinction event on the abundance of metazoans caused a relaxation of ecological constraints and consequently allowed microbialite‐generating microbial mats to flourish. A significant reduction in metazoan abundance would have led to a lack of competition for space and resources during the Griesbachian and would explain why microbialites were able to develop. Previous studies also attributed the proliferation of Spathian microbialites to the loss of ecosystem engineers (such as grazers) that are inferred to limit microbialite development (Schubert and Bottjer, 1992; Pruss and Bottjer, 2004). Grazers are, however, present within the P/Tr boundary microbialites and, therefore, must not have been abundant enough to limit microbialite development. Furthermore, this explanation may demonstrate why the first phase of microbialite proliferation ended in the late Griesbachian and Dienerian: competing shallow marine benthic communities from this interval are interpreted to represent initial recovery from the mass extinction (Hofmann *et al.*, 2011; Foster *et al.*, 2017). The keratose sponges recorded within the microbialites are interpreted as r‐strategists, and during ‘background times’ they also occur in association with microbialites and are limited to settings associated with less favourable conditions (Luo and Reitner, 2014). The presence of keratose sponges in the absence of microbialites in central Iran and Armenia at the P/Tr boundary (Leda *et al.*, 2014; Friesenbichler *et al.*, 2018) suggests that they do not require the close association of microbialites. Instead, competition with other species limits the distribution of keratose sponges, as is also observed for protomonaxoid sponges (Brayard *et al.*, 2017). Herein, it is inferred that competitive exclusion was the main control on the presence or absence of microbialites. This is because ooids, which also require carbonate supersaturated waters (Lehrmann *et al.*, 2012; Li *et al.*, 2013, 2019), are abundant for most of the Early Triassic, even in the absence of microbialites during competitive times with metazoans.

This study focused on the development of microbialites around the P/Tr boundary and does not consider subsequent phases of microbialite development during the Early Triassic or controls on the development of microbialites from the Panthalassa Ocean. These subsequent phases of microbialite development during the Early Triassic have been related to additional biotic crises (see Baud *et al.*, 2007), and previous studies of the Spathian microbialites from eastern Panthalassa also show that they developed with metazoans in an oxygenated setting (Schubert and Bottjer, 1992; Marenco *et al.*, 2012) consistent with an ecological rather than an environmental control on their development.

## 6 CONCLUSIONS

The distribution and faunal composition of P/Tr boundary microbialite successions were investigated in Hungary, Slovenia, central Iran, south‐west Iran, Armenia, Turkey and South China. The Neotethyan sections record unique metazoan associations dominated by keratose sponges within a microbial framework. These also include metazoans intolerant of low‐oxygen conditions. Contrary to previous studies, these microbialite successions are interpreted to have developed in wave‐influenced, oxygenated environments. The metazoans within the microbialites are allochthonous and most likely did not live within a microbial mat. Therefore, it is suggested that (a) suppressed competition, as a result of the mass extinction event, allowed microbialite‐forming microbial mats to flourish and (b) that carbonate supersaturation in seawater favoured widespread microbialite formation following the end‐Permian mass extinction.

## CONFLICT OF INTEREST

The authors have no conflict of interest to declare.

## Data Availability

The data that support the findings of this study are available in the Supporting information.
