# Supplementary material for: Suppressed competitive exclusion enabled the proliferation of Permian/Triassic boundary microbialites
Source: Depos Rec. 2019 Nov 20;6(1):62–74. doi: 10.1002/dep2.97 (PMC7043383; doi:10.1002/dep2.97)
Supplement: Supplementary file 1 [file DEP2-6-62-s001.zip › dep297-sup-0001-Suppinfo.docx]

Suppressed competitive exclusion enabled the proliferation of Permian/Triassic boundary microbialites

**William J. Foster, Katrin Heindel, Sylvain Richoz, Jana Gliwa, Daniel J. Lehrmann, Aymon Baud, Tea Kolar-Jurkovšek, Dunja Aljinović, Bogdan Jurkovšek, Dieter Korn, Rowan C. Martindale, and Jörn Peckmann**

**Supplementary Material**

**
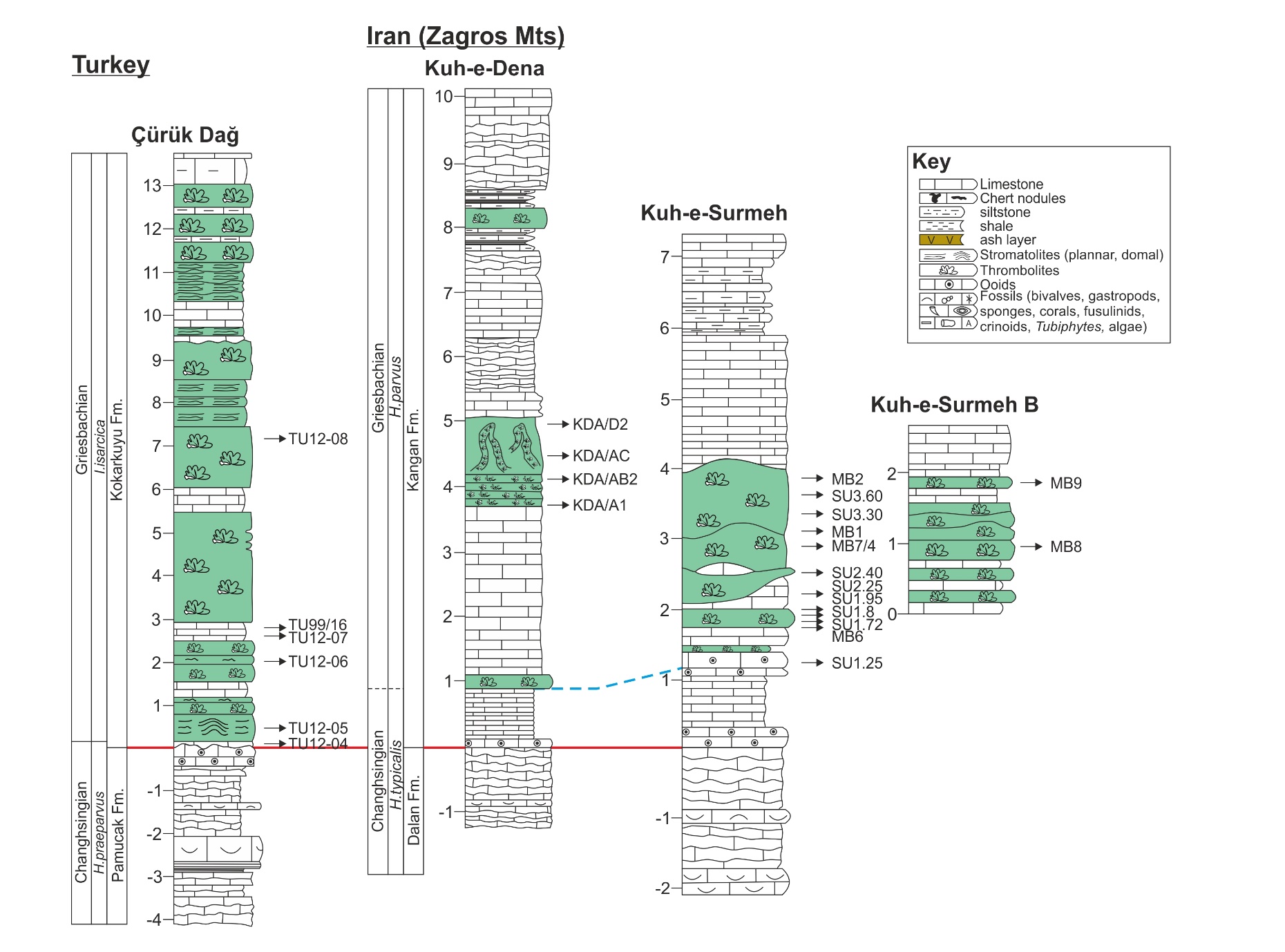

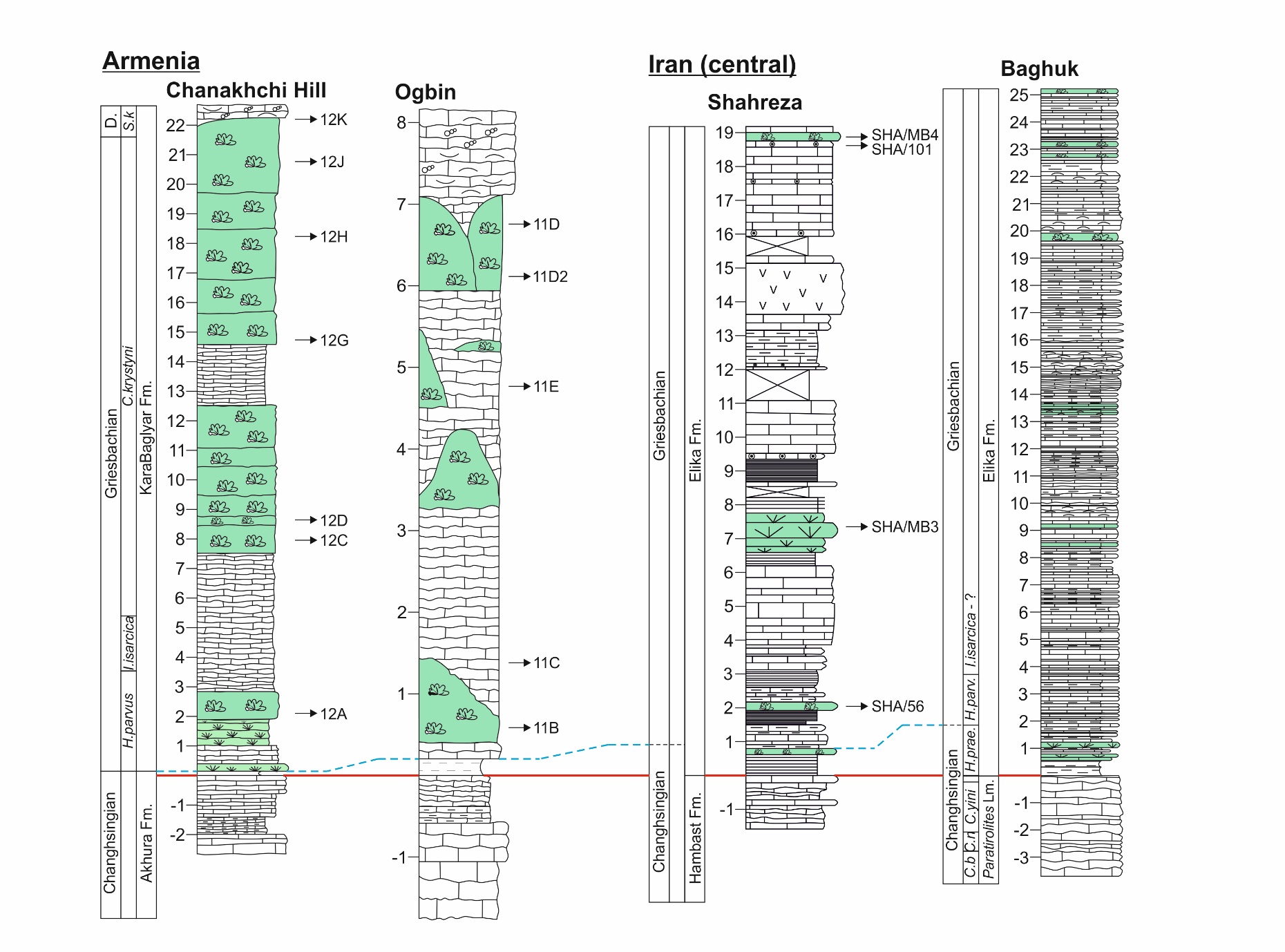

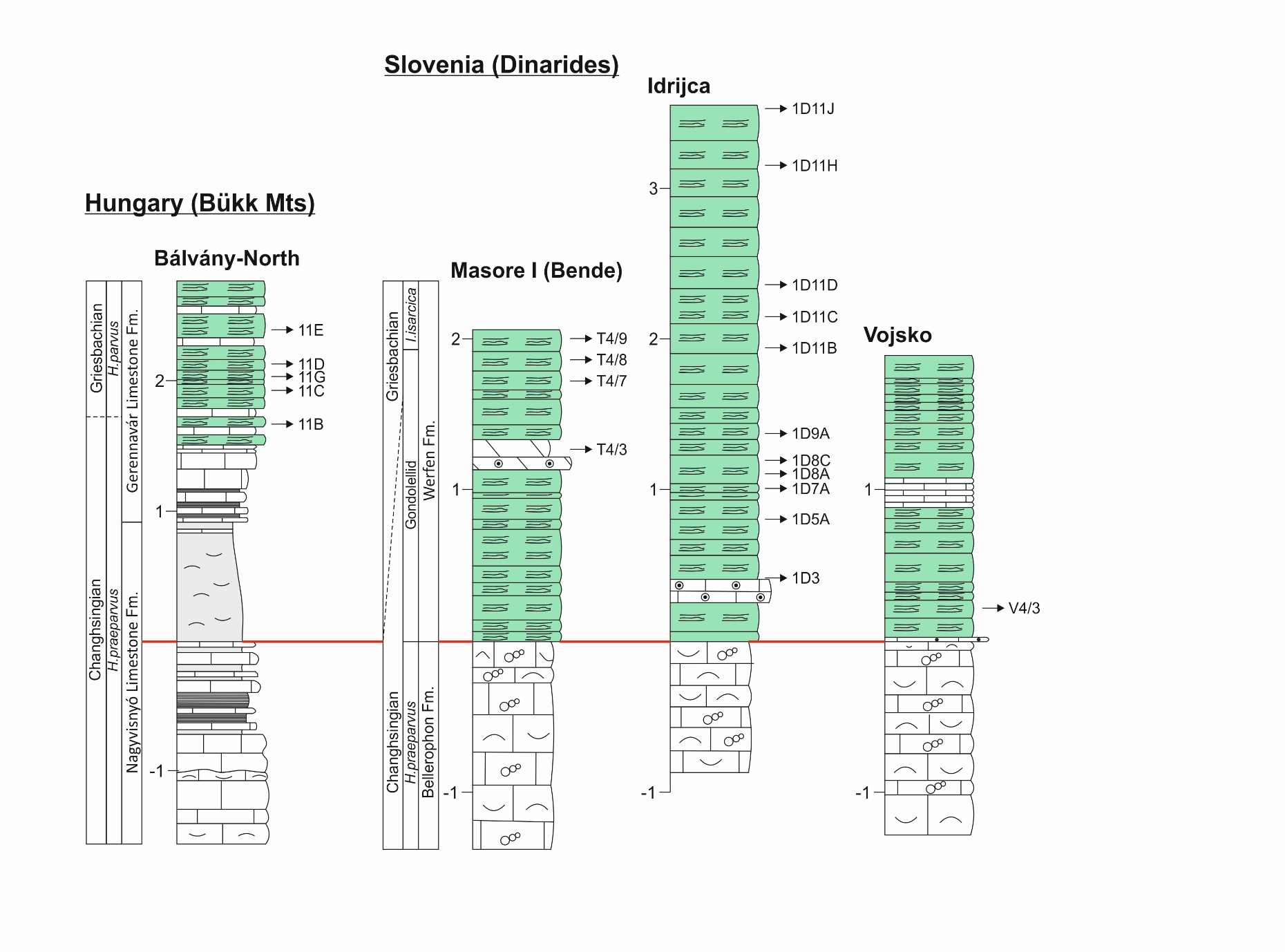

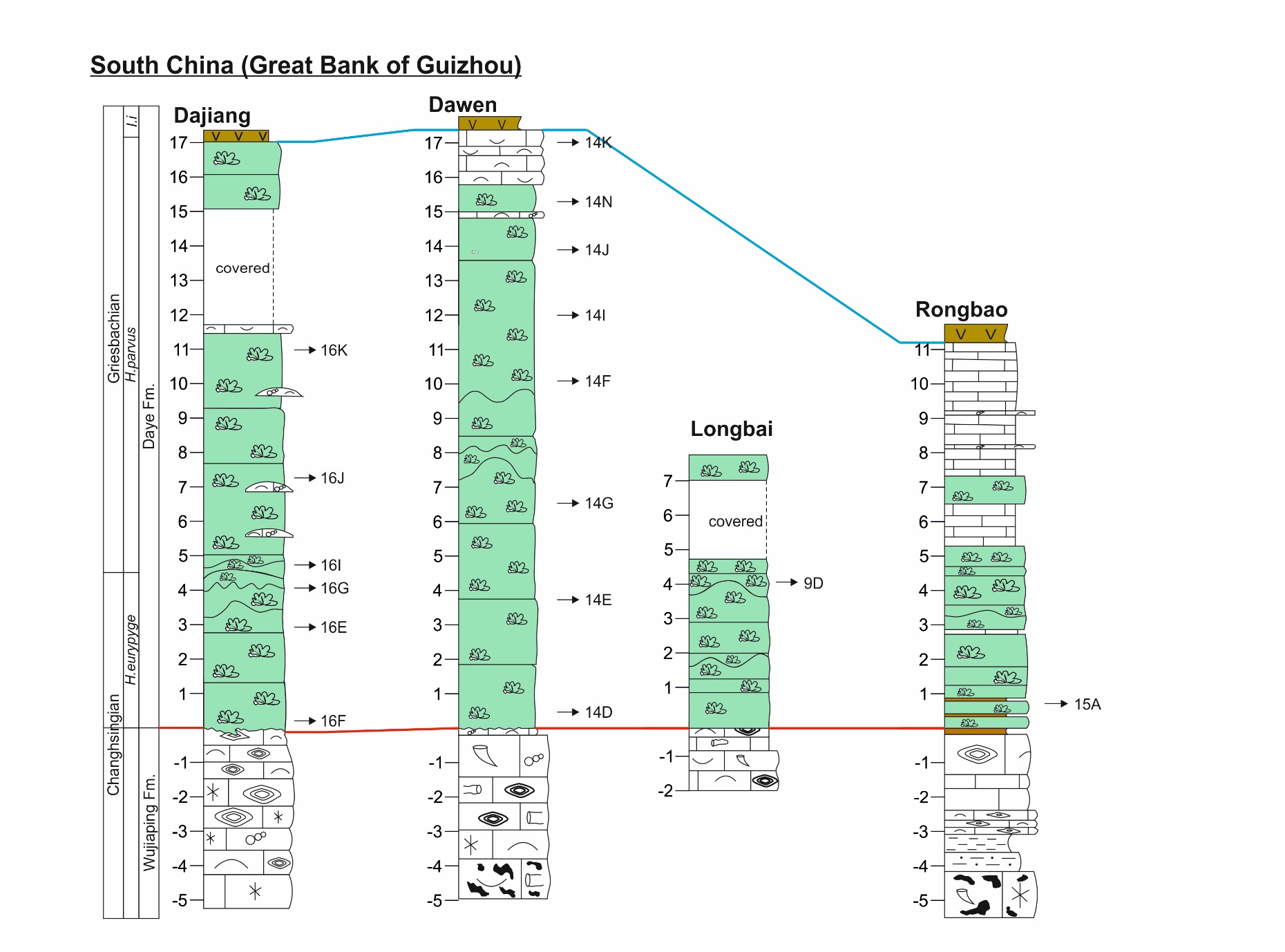

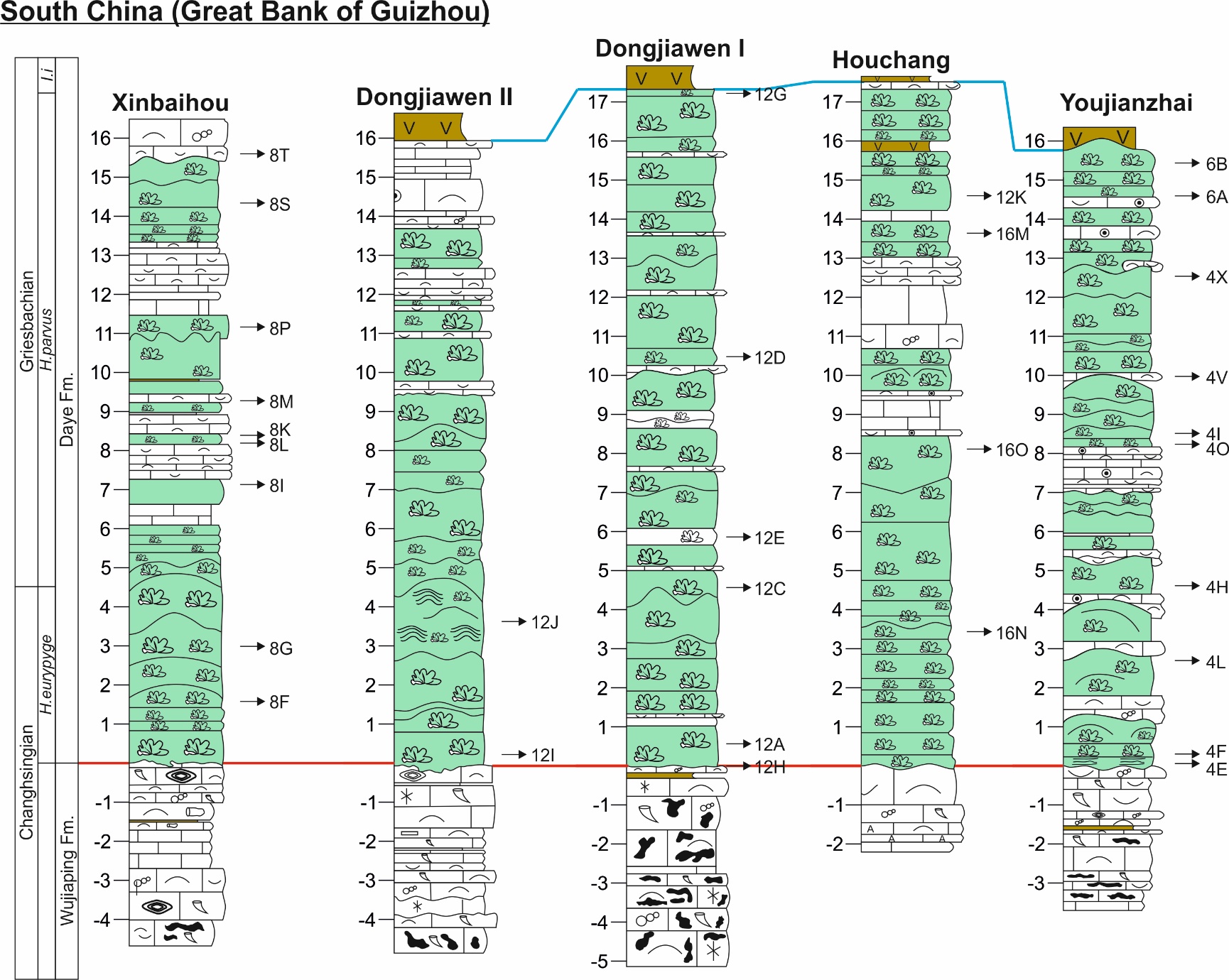
**

**←Fig. S1. Sedimentary logs of the investigated Permian/Triassic boundary microbialite sections.** Conodont zones for: Çürük Dag and Kuh-e-Surmeh after Heindel et al. (2018), Kuh-e-Dena after (Heindel et al., 2015), Chanakhchi Hill and Ogbin after (Zakharov et al., 2005) and (Sahakyan et al., 2017), Shahreza after (Richoz, 2006; Baud et al., In Prep), Baghuk after (Farshid et al., 2016), Bálvány-North after (Sudar et al., 2008), Slovenia after (Kolar-Jurkovšek et al., 2018), and the Great Bank of Guizhou after (Chen et al., 2009). Beds that record microbialites and carbonate crystal fans are highlighted in green. The extinction horizon that correlates between the sections is heighted by a solid horizontal red line. The Permian/Triassic boundary is correlated by a horizontal blue dashed line.

**
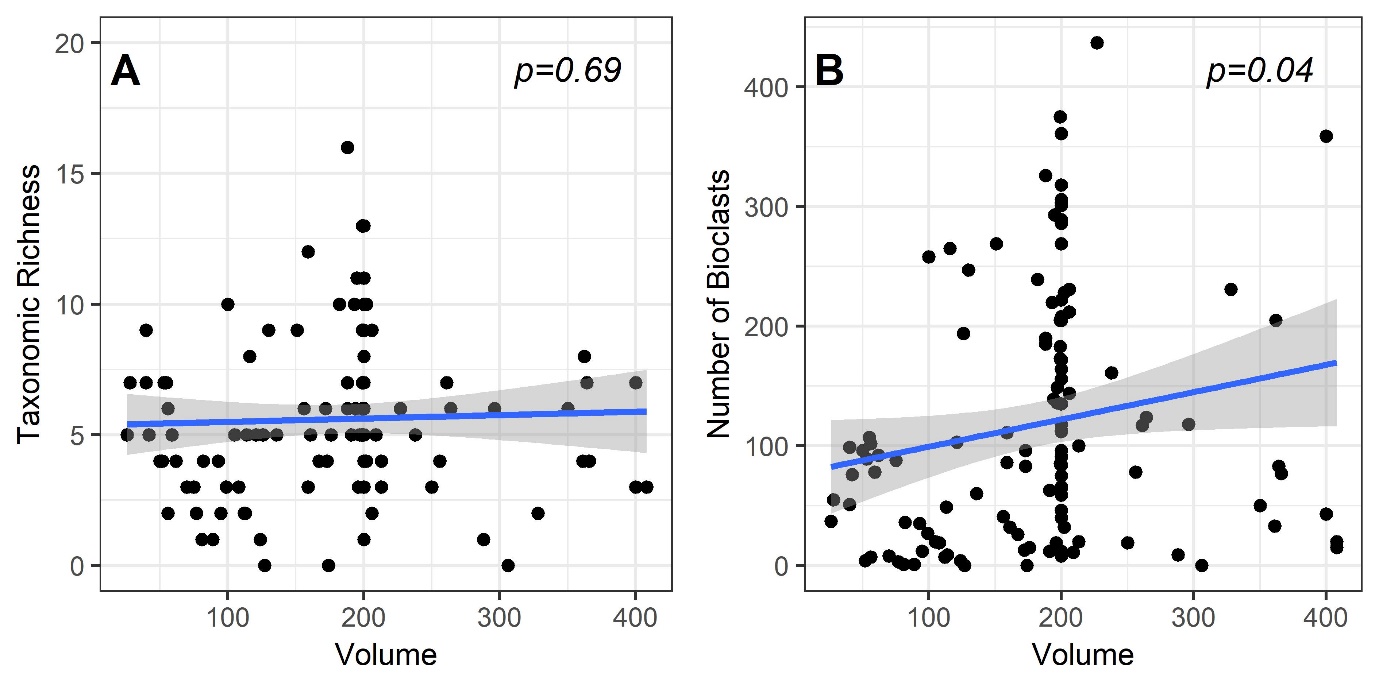
**

**Fig. S2. Relationship between the volume of investigated rock (number of 5 x 5 mm quadrats) shown by a regression line and 95% confidence intervals for both (A) Taxonomic Richness and (B) Number of Bioclasts.**

**References**

**Baud, A., Richoz, S., Brandner, R., Krystyn, L., Heindel, K., Mohtat, T. & Mohtat-Aghai, P.** (In Prep) Sponge microbial buildup: a new view on the enigmatic basal Triassic crystal layer records from Central Iran.

**Chen, J., Beatty, T.W., Henderson, C.M. and Rowe, H.** (2009) Conodont biostratigraphy across the Permian–Triassic boundary at the Dawen section, Great Bank of Guizhou, Guizhou Province, South China: Implications for the Late Permian extinction and correlation with Meishan. *Journal of Asian Earth Sciences*, **36,** 442-458.

**Farshid, E., Hamdi, B.A.-D., Hirapatani, W. and Aqanbati, S.A.** (2016) Conodont biostratigraphy of the Permian-Triassic boundary in the baghuk mountain section Northwest of Abadeh. *Geosciences*, **25,** 285-294.

**Heindel, K., Richoz, S., Birgel, D., Brandner, R., Klügel, A., Krystyn, L., Baud, A., Horacek, M., Mohtat, T. and Peckmann, J.** (2015) Biogeochemical formation of calyx-shaped carbonate crystal fans in the subsurface of the Early Triassic seafloor. *Gondwana Research*, **27,** 840-861.

**Kolar-Jurkovšek, T., Jurkovšek, B., Nestell, G.P. and Aljinović, D.** (2018) Biostratigraphy and sedimentology of Upper Permian and Lower Triassic strata at Masore, Western Slovenia. *Palaeogeography, Palaeoclimatology, Palaeoecology*, **490,** 38-54.

**Richoz, S.** (2006) Stratigraphie et variations isotopiques du carbone dans le Permien supérieur et le Trias inférieur de quelques localités de la Néotéthys (Turquie, Oman et Iran). *Mémoires de Géologie (Lausanne)* **46,** 1-283.

**Sahakyan, L., Baud, A., Grigoryan, A., Friesenbichler, E. and Richoy, S.** (2017) The Permian-Triassic transition in Southern Armenia. 5th IGCP 630 International conference and field workshop, 8-14 October, 2017. *National Academy of Sciences of the Armenia Republic, Institute of Geological Sciences, Yerevan, Field Guide Book,***,** 1-53.

**Sudar, M., Perri, M.C. and Haas, J.** (2008) Conodonts across the Permian-Triassic boundary in the Bükk Mountains (NE Hungary). *Geologica Carpathica*, **59,** 491-502.

**Zakharov, Y.D., Biakov, A.S., Baud, A. and Kozur, H.** (2005) Significance of Caucasian sections for working out carbon-isotope standard for Upper Permian and Lower Triassic (Induan) and their correlation with the Permian of North-Eastern Russia. *Journal of China University of Geosciences*, **16,** 141-151.
